# Impact of Radial Wall Strain on Serial Changes in Vascular Physiology in Patients with Intermediate Coronary Stenosis

**DOI:** 10.31083/j.rcm2408245

**Published:** 2023-08-24

**Authors:** Jiapeng Chu, Yan Lai, Wenwen Yan, Yian Yao, Hao Lin, Deqiang Yuan, Fan Ping, Guoqi Zhu, Zi Ye, Tongqing Yao, Fei Chen, Xuebo Liu

**Affiliations:** ^1^Department of Cardiology, Tongji Hospital, School of Medicine, Tongji University, 200065 Shanghai, China

**Keywords:** coronary artery disease, quantitative flow ratio, plaque progression, vascular imaging

## Abstract

**Background:**

Coronary biomechanical stress contributes to the plaque rupture and subsequent events. This study aimed to investigate the impact of plaque biomechanical stability on the physiological progression of intermediate lesions, as assessed by the radial wall strain (RWS) derived from coronary angiography.

**Methods:**

Patients with at least one medically treated intermediate lesion at baseline who underwent follow-up coronary angiography over 6 months were included. The maximal RWS (${\mathrm{RWS}}_{\text{max}}$) of the interrogated lesion was calculated from the baseline angiogram. The primary endpoint was to determine the association between baseline ${\mathrm{RWS}}_{\text{max}}$ and the functional progression of coronary lesions, defined as an increase in the lesion-specific △quantitative flow ratio (L-△QFR, calculated as the absolute change in QFR across the lesion) on serial angiograms.

**Results:**

Among 175 lesions in 156 patients, 63 lesions showed an increase in L-△QFR during a median follow-up period of 12.4 months. Baseline ${\mathrm{RWS}}_{\text{max}}$ values were significantly higher in lesions with increased L-△QFR than in those with stabilized or decreased L-△QFR (11.8 [10.7, 13.7] *vs*.10.8 [9.7, 11.7]; *p* = 0.001). Baseline ${\mathrm{RWS}}_{\text{max}}$ presented an area under the curve of 0.658 (95% confidence interval [CI]: 0.572–0.743, *p* < 0.001) for the prediction of increased L-△QFR. After full adjustment for clinical and angiographic factors, a high ${\mathrm{RWS}}_{\text{max}}$ (>12) was found to be an independent predictor of functional lesion progression (odds ratio: 2.871, 95% CI: 1.343–6.138, *p* = 0.007).

**Conclusions:**

A high ${\mathrm{RWS}}_{\text{max}}$ calculated from baseline angiograms was independently associated with the subsequent physiological progression in patients with intermediate coronary lesions.

## 1. Introduction

Recent advances in coronary plaque imaging have led to an increased interest in detecting and treating of vulnerable plaque features that are associated with the risk of future cardiovascular events. A vulnerable coronary plaque is typically characterized by a thin fibrous cap and large lipid core with abundant inflammatory cells, which can be visualized using non-invasive or invasive coronary imaging modalities [1]. Although a number of imaging studies have provided important insights by showing that the geometrical and morphological features of vulnerable plaques are of clinical significance (i.e., associated with lesion progression and clinical outcomes), their relatively low positive predictive value for patient prognosis indicates additional information is needed to extend our knowledge of the “high-risk” plaques [2, 3]. In this context, coronary strain, which can be measured using intravascular elastography or palpography, has been proposed as a valuable biomechanical method to assess the plaque vulnerability [4, 5]. High-strain spots across the coronary lesions have been proven to correlate with an increased risk of acute coronary syndrome [5]. However, the conventional method of strain analysis is cumbersome, which hinders the adoption of biomechanical assessment in clinical practice. To address this unmet need, Hong *et al*. [6] introduced a simplified artificial intelligence-aided method of calculating coronary strain, labelled as radial wall strain (RWS), from conventional coronary angiography (CAG). They also demonstrated that angiography-derived RWS correlates well with the validated characteristics of vulnerable plaques by optical coherence tomography (OCT), indicating that RWS could be a less-invasive alternative tool for evaluating plaque vulnerability from a biomechanical aspect.

Plaque progression, usually measured by the serial change in the severity of luminal stenosis on angiography or atheroma volume on intracoronary imaging, has been acknowledged as a necessary and modifiable step between early atherosclerosis and acute coronary events [7]. Beyond the anatomic parameters, coronary physiology is also an important aspect of plaque characteristics, which has independent significance on clinical outcomes [8]. In particular, recent studies have revealed that pressure wire-based or angiography-derived physiological indices could be a surrogate marker for evaluating the functional change of coronary lesions, and for monitoring the effectiveness of a certain anti-atherosclerotic therapy [9, 10, 11].

The present study aimed to investigate the association between the biomechanical stability of coronary plaques as assessed by angiographic RWS and disease progression in coronary physiology as evaluated by serial quantitative flow ratio (QFR).

## 2. Materials and Methods

### 2.1 Study Population

**Fig. 1.**
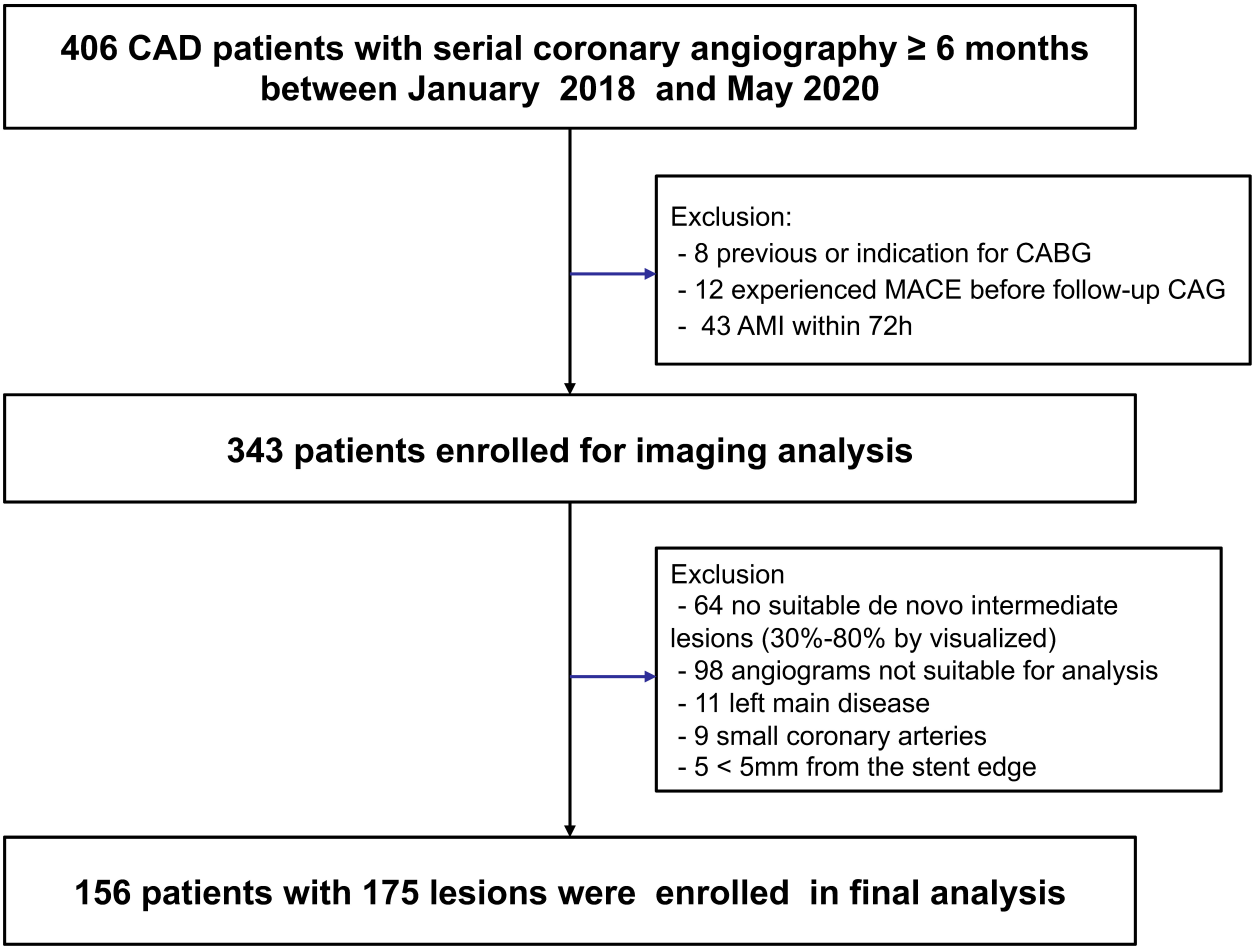
**Study flow chart**. CAD, coronary artery disease; CABG, coronary artery bypass graft; MACE, major adverse cardiac events; CAG, coronary angiography; AMI, acute myocardial infarction.

This retrospective cohort study was performed at Tongji Hospital, Tongji University, Shanghai. Patients with suspended or known coronary artery disease (CAD) who underwent serial CAG examinations at intervals of ≥6 months between January 2018 and May 2020 were retrospectively enrolled (Fig. 1). Patients were excluded if they had an acute myocardial infarction within 72 h, had previous or planned coronary artery bypass graft surgery, or experienced adverse cardiac events in between the two CAG measurements. For inclusion, the target lesions had to meet the following criteria at baseline angiography: (1) de novo lesions that were not considered for interventional procedures or stent implantation, (2) intermediate lesions with a percentage diameter stenosis between 30% and 80%, and (3) lie in a vessel with a reference diameter ≥2 mm by visual estimation. Angiographic exclusion criteria included significant left main disease, small coronary arteries, in-stent restenosis within 5 mm of the stent edge, inadequate angiographic image quality or other reasons limiting the computations of QFR or RWS. This study was conducted in accordance with the Declaration of Helsinki. The Ethics Committee of Tongji Hospital, Tongji University, approved the study protocol.

### 2.2 QFR and Angiographic Analysis 

Angiographic images were obtained according to the standard of care based on local practice. The decision to perform percutaneous coronary intervention (PCI) during the procedure was at the discretion of the treating physician. Serial Murray-law based QFR and quantitative coronary angiography (QCA) analyses were performed at index and follow-up CAG procedures using a QFR system software (AngioPlus Core version 2.0, Pulse Medical, Shanghai, China), as previously described [12]. QFR and QCA data including minimum lumen diameter, reference diameter, lesion length, and percent diameter stenosis (DS%) were routinely obtained from the software. In addition to the computation of the traditional QFR value of the entire vessel, we measured the absolute change in QFR across the interrogated lesion (named as lesion-specific △QFR, L-△QFR), a more accurate index for quantifying hemodynamic significance driven by a specific lesion.

### 2.3 RWS Analysis 

Offline RWS was measured using a recently developed software (AngioPlus Core version 3.0, Pulse Medical, Shanghai, China) by experienced analysts who were blinded to the serial QFR results. The detailed theory and procedures for RWS analysis have been described and validated by previous studies, and RWS is defined as the relative diameter deformation over the cardiac cycle for each position [6, 13]. In brief, one high-quality angiographic projection at the end-diastole was selected and transferred to software by the analyst. Another 3 frames at different periods of the cardiac cycle were automatically selected by the software. Subsequently, the lumen contours of the interrogated vessels were automatically delineated in the four selected frames and thus depicting a map of the lumen diameters along the interrogated segments throughout the cardiac cycle. In the present study, the RWS was calculated along the interrogated lesions, and the maximum RWS (${\mathrm{RWS}}_{\text{max}}$) was defined as the lesion RWS. An ${\mathrm{RWS}}_{\text{max}}$ >12 was categorized as high as described previously [6]. Inter- and intra-observer reproducibility of ${\mathrm{RWS}}_{\text{max}}$ was assessed in 50 randomly selected lesions. The intraclass correlation coefficients were 0.872 (95% confidence interval [CI], 0.786–0.925) for inter-observer variability and 0.936 (95% CI, 0.890–0.963) for intra-observer variability, suggesting good agreement between the analysts.

### 2.4 Data Collection and Study Endpoint 

All clinical data during the baseline procedure, including patient demographics, clinical presentation, conventional risk factors, blood tests, and medical treatments, were collected by reviewing the hospital database. Diabetes mellitus was defined as glycosylated hemoglobin ≥6.5%, fasting plasma glucose ≥7.0 mmol/L or 2 h plasma glucose ≥11.1 mmol/L [14]. The estimated glomerular filtration rate (eGFR) was calculated using the modified Diet in Renal Disease equation [15]. The primary objective of the current study was to explore the impact of plaque vulnerability evaluated by the RWS on the hemodynamic change of the lesion, and thus we selected the lesion-level functional progression (FP), defined as an increase in L-△QFR (L-△QFR at follow-up minus L-△QFR at baseline >0) value, as the primary endpoint.

### 2.5 Statistical Analysis 

Categorical variables are expressed as absolute counts and percentages and compared between the groups using Pearson’s chi-square or Fisher’s exact test. Continuous variables are presented as mean ± SD or median with a 25% to 75% interquartile range, and compared between groups using Student’s *t* tests, Wilcoxon signed rank test, and the Mann–Whitney *U* test, as appropriate. For the lesion-level-based analysis, generalized linear mixed-effects logistic regression models were used to assess the predictive value of baseline clinical or angiographic parameters and RWS for functional lesion progression. Patient identification was included as a random effect to account for the nonindependence of lesions within the same patient. Univariable and multivariable models were derived to obtain odds ratios (ORs) and 95% CIs. Adjustments were made for variables including age, sex, the time interval between the CAG measurements, and other baseline clinical factors or angiographic parameters with a *p*-value < 0.10 (acute coronary syndrome; diabetes mellitus; eGFR, PCI in interrogated vessels and vessel QFR) in the univariable analyses. The predictive ability of baseline ${\mathrm{RWS}}_{\text{max}}$ and DS% for increased L-△QFR values were quantified using the receiver-operating characteristic (ROC) curve and the area under the curve (AUC) by using the Delong method. A 2-tailed *p*-value < 0.05 was considered statistically significant. All statistical analyses were performed using SPSS version 26.0 (SPSS, IBM Corp., Armonk, NY, USA) and R version 3.6.1 (R Foundation for Statistical Computing, Vienna, Austria).

## 3. Results

A total of 175 intermediate de novo lesions from 156 patients who underwent serial angiography were enrolled in the present study. Follow-up CAG measurements were performed at a median time interval of 12.4 (10.9, 16.6) months, after the index procedures. The baseline patient characteristics are shown in Table 1. The mean age was 65.2 ± 9.4 years, and most patients (69.2%) were male. The median L-△QFR for the enrolled 175 lesions was 0.04 (0.02, 0.06) at baseline and slightly increased to 0.05 (0.03, 0.07) at follow-up. Out of these, 63 (36%) lesions showed an increase in the L-△QFR value (FP group), whereas 112 (64%) lesions remained the same or decreased (non-FP group). The lesion and procedural characteristics of the two groups are shown in Table 2. At baseline, lesions with FP showed a higher proportion of PCI in the interrogated vessels than in the non-FP group. No significant differences were observed in baseline lesion location, QCA parameters, or vessel or lesion-specific QFR values between the two groups. However, at follow-up angiography, DS% and L-△QFR were significantly greater, while the minimal diameter and vessel QFR were statistically lower in lesions with FP. Importantly, we revealed that baseline ${\mathrm{RWS}}_{\text{max}}$ levels were significantly higher in the lesions with increased L-△QFR than in controls (11.8 [10.7, 13.7] *vs*.10.8 [9.7, 11.7], *p* = 0.001).

**Table 1.** **Baseline patient characteristics**.

Patient characteristics (N = 156)		
Follow-up period, months		12.4 (10.9, 16.6)
Age, years		65.2 ± 9.4
Male (%)		108 (69.2)
Body mass index, ${\mathrm{kg/m}}^{2}$		24.3 ± 3.0
Hypertension (%)		105 (67.3)
Diabetes mellitus (%)		63 (40.4)
Hyperlipidemia (%)		104 (66.7)
Previous or current smoker (%)		87 (55.8)
Previous myocardial infarction (%)		23 (14.7)
Previous stroke		13 (8.3)
Clinical presentation		
	Stable coronary artery disease (%)	95 (60.9)
	${\mathrm{Acute coronary syndrome}}^{*}$ (%)	61 (39.1)
Medical treatments at discharge		
	Statins (%)	152 (97.4)
	β-blockers (%)	107 (68.6)
	ACEI or ARB (%)	96 (61.5)
	Dual antiplatelet therapy (%)	106 (67.9)
Laboratory data		
	high sensitivity C-reactive protein, mg/L	2.12 (0.81, 4.75)
	Estimated glomerular filtration rate, mL/min/1.73 $m^{2}$	82.1 (71.8, 94.8)
	Total cholesterol, mmol/L	4.29 (3.65, 5.24)
	Triglycerides	1.46 (1.11, 2.09)
	High-density lipoprotein cholesterol, mmol/L	1.00 (0.88, 1.26)
	Low-density lipoprotein cholesterol, mmol/L	2.90 (2.24, 3.55)
	Glycosylated hemoglobin, %	6.2 (5.8, 6.9)

Data are expressed as n (%), mean ± SD, or median (25th, 75th percentiles). ACEI, angiotensin-converting enzyme inhibitors; ARB, angiotensin II receptor antagonists. *Acute coronary syndrome includes patients with unstable angina and myocardial infarction within 30 days of procedure.

**Table 2.** **Comparison of angiographic and physiological characteristics**.

Variable		Total (N = 175)	Non-FP (N = 112)	FP (N = 63)	*p*-value
Location of culprit lesion (%)					0.433
	Left anterior descending artery	55 (31.4)	33 (29.5)	22 (34.9)	
	Left circumflex artery	42 (24.0)	25 (22.3)	17 (27.0)	
	Right coronary artery	78 (44.6)	54 (48.2)	24 (38.1)	
PCI in interrogated vessels (%)		14 (8.0)	5 (4.5)	9 (14.3)	0.022
Baseline ${\mathrm{RWS}}_{\text{max}}$		11.2 (9.9, 12.5)	10.8 (9.7, 11.7)	11.8 (10.7, 13.7)	<0.001
Baseline ${\mathrm{RWS}}_{\text{max}}$ >12 (%)		53 (30.3)	23 (20.5)	30 (47.6)	<0.001
Baseline angiographic and physiological parameters					
	Minimal diameter, mm	2.1 (1.8, 2.4)	2.2 (1.9, 2.5)	2.1 (1.8, 2.4)	0.123
	Reference diameter, mm	3.4 (3.0, 3.6)	3.4 (3.0, 3.6)	3.3 (2.8, 3.6)	0.179
	Diameter stenosis, %	35 (29, 40)	34 (29, 39)	35 (30, 40)	0.474
	Lesion length, mm	16.3 (12.2, 23.2)	17.3 (12.5, 22.5)	14.6 (11.7, 22.0)	0.179
	Vessel QFR	0.94 (0.90, 0.96)	0.94 (0.90, 0.96)	0.93 (0.89, 0.95)	0.065
	Lesion-specific △QFR	0.04 (0.02, 0.06)	0.04 (0.02, 0.06)	0.04 (0.03, 0.06)	0.902
Follow-up angiographic and physiological parameters					
	Minimal diameter, mm	2.1 (1.8, 2.4)	2.2 (2.0, 2.5)	1.9 (1.6, 2.3)	<0.001
	Reference diameter, mm	3.2 (3.0, 3.6)	3.3 (3.0, 3.5)	3.2 (2.9, 3.6)	0.463
	Diameter stenosis, %	35 (29, 42)	32 (27, 39)	41 (35, 45)	<0.001
	Lesion length, mm	18.4 (13.1, 23.5)	17.8 (12.5, 22.8)	19.1 (13.5, 25.3)	0.177
	Vessel QFR	0.93 (0.88, 0.95)	0.94 (0.91, 0.96)	0.89 (0.84, 0.93)	<0.001
	Lesion-specific △QFR	0.05 (0.03, 0.07)	0.03 (0.02, 0.05)	0.06 (0.05, 0.09)	<0.001

Data are expressed as n (%) or median (25th, 75th percentiles). FP, functional progression; PCI, percutaneous coronary intervention; RWS, radial wall strain; ${\mathrm{RWS}}_{\text{max}}$, maximum radial wall strain; QFR, quantitative flow ratio.

**Fig. 2.**
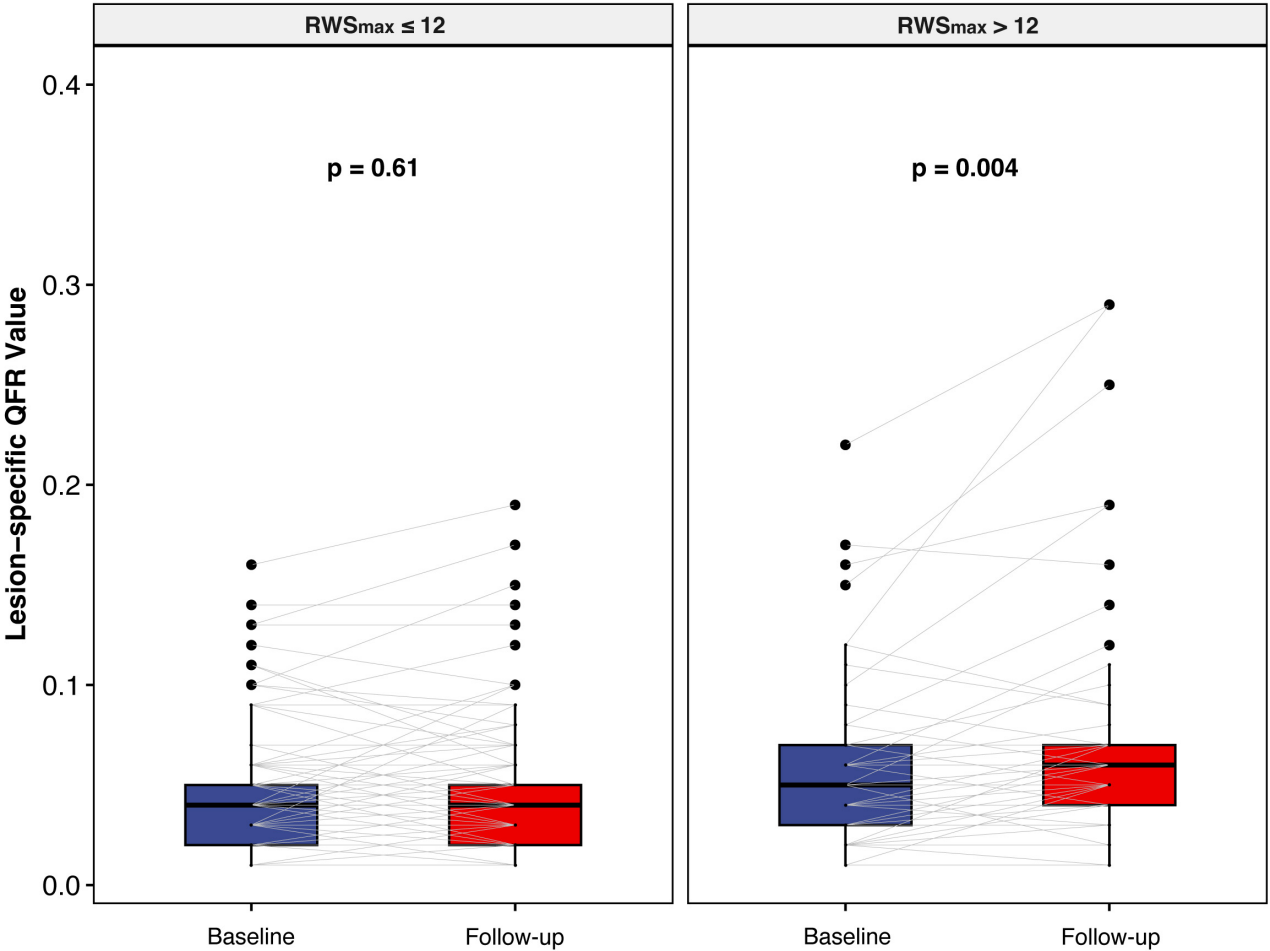
**Changes in lesion-specific QFR from index to follow-up procedures according to baseline ${\mathrm{RWS}}_{\text{𝐦𝐚𝐱}}$ levels**. RWS, radial wall strain; ${\mathrm{RWS}}_{\text{max}}$, maximum radial wall strain; QFR, quantitative flow ratio.

According to the established cut-off value, lesions were divided into high (${\mathrm{RWS}}_{\text{max}}$ >12) and low (${\mathrm{RWS}}_{\text{max}}$ ≤12) strain groups. The angiographic and procedural characteristics stratified by ${\mathrm{RWS}}_{\text{max}}$ are shown in **Supplementary Table 1**. An ${\mathrm{RWS}}_{\text{max}}$ >12 was more frequently presented in lesions with subsequent functional progression (47.6% *vs*. 20.5%, *p* < 0.001). The change in the L-△QFR value from baseline to follow-up according to the baseline ${\mathrm{RWS}}_{\text{max}}$ is shown in Fig. 2. In lesions with high baseline ${\mathrm{RWS}}_{\text{max}}$ (>12), the L-△QFR value was 0.05 (0.03, 0.07) at baseline and slowly increased to 0.06 (0.04, 0.07) at follow-up (*p *= 0.004), while no statistical difference was observed in lesions with baseline ${\mathrm{RWS}}_{\text{max}}$ ≤12 (0.04 [0.02, 0.05] *vs*. 0.04 [0.02, 0.05], *p* = 0.61).

**Fig. 3.**
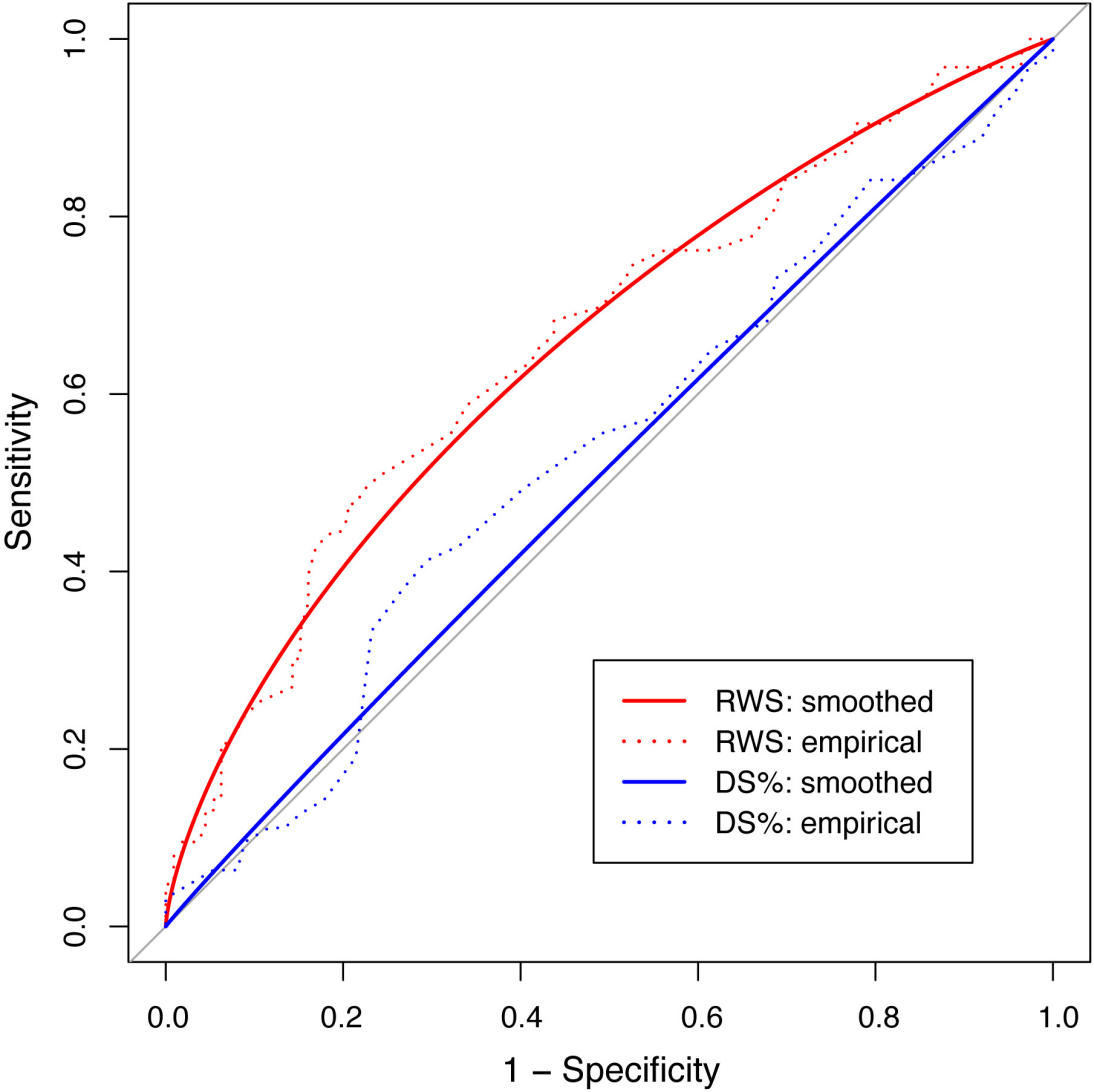
**Receiver operator curves of baseline ${\mathrm{RWS}}_{\text{𝐦𝐚𝐱}}$ and DS% for predicting increase of lesion-specific △QFR**. The red line is baseline ${\mathrm{RWS}}_{\text{max}}$ (AUC: 0.658, 95% CI: 0.572–0.743, *p* < 0.001); the blue line is baseline DS% (AUC, 0.533, 95% CI: 0.442–0.623; *p* = 0.763). AUC, area under curve; CI, confidence interval; RWS, radial wall strain; DS, diameter stenosis; RWS, radial wall strain; ${\mathrm{RWS}}_{\text{max}}$, maximum radial wall strain; QFR, quantitative flow ratio.

Univariable generalized linear mixed-effects regression analysis showed that ${\mathrm{RWS}}_{\text{max}}$ was associated with a high risk of subsequent functional lesion progression (unadjusted OR: 1.267, 95% CI: 1.105–1.453, *p* = 0.001) (**Supplementary Table 2**). Moreover, the significant predictive value of the baseline ${\mathrm{RWS}}_{\text{max}}$ was preserved after adjustment for traditional cardiovascular risk factors and angiographic characteristics using different models. The results were consistent when considering a high ${\mathrm{RWS}}_{\text{max}}$ (>12) as a categorical variable to evaluate its relationship with functional lesion progression (Table 3). Furthermore, we plotted the ROC curves of baseline ${\mathrm{RWS}}_{\text{max}}$ and DS% to predict a subsequent lesion-level FP. As shown in Fig. 3, the AUC was significantly greater for ${\mathrm{RWS}}_{\text{max}}$ than for DS% (0.658 [0.572–0.743] *vs*. 0.533 [0.442–0.623], *p* = 0.021).

**Table 3.** **Generalized linear mixed-effects logistic regression analyses of ${\mathrm{RWS}}_{\text{𝐦𝐚𝐱}}$ for the increase of lesion-specific △QFR**.

	1-U increase in ${\mathrm{RWS}}_{\text{max}}$			${\mathrm{RWS}}_{\text{max}}$ >12		
	OR	95% CI	*p*-value	OR	95% CI	*p*-value
Unadjusted	1.267	1.105–1.453	0.001	3.617	1.823–7.176	<0.001
Model 1	1.270	1.106–1.458	0.001	3.678	1.835–7.374	<0.001
Model 2	1.269	1.099–1.464	0.001	3.317	1.609–6.838	0.001
Model 3	1.237	1.066–1.436	0.005	2.871	1.343–6.138	0.007

Values are presented as ORs (with 95% CIs) derived via generalized linear mixed-effects logistic regression analysis. Model 1: adjusted for age, sex, interval time between CAG measurements. Model 2: Model 1 + adjusted for clinical risk factors (acute coronary syndrome; diabetes mellitus; eGFR); Model 3: model 2 + adjusted for angiographic risk factors (PCI in interrogated vessels and baseline vessel QFR). RWS, radial wall strain; ${\mathrm{RWS}}_{\text{max}}$, maximum radial wall strain; OR, odds ratio; CI, confidence interval; CAG, coronary angiography; eGFR, estimated glomerular filtration rate; PCI, percutaneous coronary intervention; QFR, quantitative flow ratio.

## 4. Discussion

In the current study, we used a novel angiography-based approach to quantify the RWS in intermediate coronary lesions and investigated the impact of focal plaque strain on the longitudinal changes in coronary physiology. The major findings were as follows: lesions with a higher ${\mathrm{RWS}}_{\text{max}}$ (>12) at baseline were associated with a higher rate of subsequent functional lesion progression as defined by an increase in the L-△QFR value, and elevated baseline ${\mathrm{RWS}}_{\text{max}}$ levels were found to be an independent predictor of functional progression in the multivariable-adjusted models.

Palpography and elastography using intravascular imaging are the major methods to evaluate coronary strain by detecting differences in the deformability of the vessel wall [4, 16, 17]. However, the clinical application of biomechanical assessment is limited because of the invasive nature of intracoronary imaging and the complexity of the computational methodology. In this regard, Hong *et al*. [6] proposed a simplified parameter labelled radial wall strain to assess the local mechanical properties of coronary plaques. This novel method can be widely applied in biomechanical assessment because it can be easily obtained from the routine coronary angiograms. In the present study, we analyzed the plaque strain using this angiography-derived RWS at baseline and monitored the dynamic change in hemodynamic status at a median follow-up of 1 year. Our findings showed that lesions with high baseline RWS presented an accelerated functional progression rate, whereas low RWS is associated with plaque stabilization. To the best of our knowledge, this is the first study demonstrated that biomechanical stress plays an important role in the functional progression of medically treated coronary lesions, which may provide clinical evidence linking the high plaque strain and the unfavorable outcomes for CAD patients.

The clinical significance of changes in coronary physiology has been investigated by several wire-based or imaging-derived fractional flow reserve (FFR) parameters [9, 10, 11, 18, 19]. As reported in a previous study, the longitudinal physiological progression is slow, as evaluated by per-vessel FFR with a median decrease of 0.007 per year [9]. A recently published study revealed that the lesion-specific computed tomography-derived FFR value was not significantly different over a mean interval of 13.9 months in patients with intermediate coronary stenosis [10]. In line with these studies, our results showed that vessel-level QFR deteriorated by 0.01 [0, 0.02] over a period of 1 year. In parallel, the overall lesion-specific QFR values also showed a slow progression rate (from 0.04 [0.02, 0.06] at baseline to 0.05 [0.03, 0.07] at follow-up). Despite coronary lesions progressing at a very slow rate in functional status, several studies, including a prospective trial, have demonstrated that intensive statin treatment could improve the hemodynamic status assessed by invasive or computational FFR [10, 11, 19], indicating that serial changes in coronary physiology could be a surrogate marker for monitoring the effect of medical treatments in patients with CAD. In this study, we further demonstrated that angiography-based QFR allowed the assessment of changes in coronary physiology of intermediate lesions and could be a useful quantitative index to evaluate coronary functional progression.

Previous studies have reported that high radial strain is associated with vulnerable plaque features and a worse prognosis in patients with CAD [5, 20]. However, the underlying mechanism remains unclear. The intracoronary imaging studies demonstrated that the major causes of acute coronary syndrome are plaque rupture and erosion, which are prone to occur at the site of high stress within the cap of a vulnerable plaque [21, 22]. Therefore, the combination of morphological vulnerability, characterized by thin fibrous caps and large lipid pools, and biomechanical vulnerability, evaluated by shear stress, plaque structural stress, or coronary strain, could improve the accuracy in the detection of high-risk plaque at high risk for adverse coronary event [22, 23, 24]. Of note, although most of the acute coronary syndromes are provoked by abrupt rupture or erosion of plaque that leads to subsequent occlusive thrombosis, recent intracoronary OCT studies have revealed that the majority of ruptured or eroded plaques remain clinically silent and experience a so-called healing process, which can be visualized as a plaque with layered phenotype on OCT images [22, 25, 26]. Furthermore, a recent OCT-based study found the layered plaque was an independent predictor of subsequent rapid lesion progression assessed by angiographic severity of the luminal narrowing, indicating silent plaque rupture and subsequent healing may be an important underlying mechanism in the development of atherosclerotic plaque [27]. Two recent studies have reported that high RWS levels are associated with an increased risk of OCT-defined vulnerable features (i.e., high lipid-to-cap ratio and the presence of thin-cap fibroatheroma) and short-term clinical outcomes, which provides the direct evidence supporting the clinical relevance between RWS and poor prognosis. Notably, the optimal threshold of ${\mathrm{RWS}}_{\text{max}}$ for identifying vulnerable plaques (12%) was consistent with that for predicting clinical outcomes [6, 28]. In the present study, we also found that ${\mathrm{RWS}}_{\text{max}}$ showed a good performance for predicting lesion progression, with the same cut-off value of 12%. Although future prospective studies are required to further validate the optimal cutoff value of RWS in various clinical scenarios, these findings indicate that RWS, a comprehensive index reflecting both biomechanical force and plaque composition, may serve as a valuable tool in the identification of high-risk plaques prone to generate rapid progression and adverse events.

## 5. Study Limitations

Several potential limitations should be taken into account. First, this was a single center, retrospective observational study with a relatively small population enrolled due to rigorous inclusion and exclusion criteria, raising concerns for possible selection bias. Second, angiographic and physiological parameters as well as coronary strain were evaluated using a computational method based on coronary angiography, the gold standard techniques, i.e., wire-based FFR, intravascular imaging and palpography, were not performed because of the retrospective nature of the study. Nevertheless, angiography-derived RWS and QFR might be the more promising tools for the comprehensive evaluation of high-risk plaques in clinical settings because of their cost-efficiency and time-saving nature. Third, there is currently no consensus regarding on the cutoff value for the definition of significant coronary functional progression. As the change in coronary physiology is a continuous variable, we defined the functional progression as any increase in lesion-specific △QFR values, which may need to be clarified by future prospective natural history studies. Finally, the clinical endpoints were not evaluated due to the small sample size. Further studies are needed to address this issue.

## 6. Conclusions

For intermediate coronary lesions treated with medication, a high RWS level derived from coronary angiography was an independent predictor of subsequent plaque progression in coronary physiology.

## Data Availability

The datasets generated and analyzed during the current study are not publicly available due to the institution policy but are available from the corresponding author on reasonable request.

## Supplementary Material

Supplementary material associated with this article can be found, in the online version, at https://doi.org/10.31083/j.rcm2408245.
