# Supplementary material for: Impact of Radial Wall Strain on Serial Changes in Vascular Physiology in Patients with Intermediate Coronary Stenosis
Source: Rev Cardiovasc Med. 2023 Aug 24;24(8):245. doi: 10.31083/j.rcm2408245 (PMC11266810; doi:10.31083/j.rcm2408245)
Supplement: Supplementary file 1 [file 2153-8174-24-8-245-s1.docx]

| **Supplementary Table 1. Comparison of Angiographic and Physiological Characteristics According to Baseline RWS_max_ levels** | | | | |
| --- | --- | --- | --- | --- |
| Variable | Total  (N = 175) | RWS_max_ ≤ 12  (N = 122) | RWS_max_ > 12  (N = 53) | *p*-value |
| Baseline RWS_max_ | 11.2 (9.9, 12.5) | 10.4 (9.2, 11.3) | 13.7 (12.9, 15.6) | < 0.001 |
| **Location of culprit lesion (%)** | | | | 0.177 |
| LAD | 55 (31.4) | 35 (28.7) | 20 (37.7) |  |
| LCX | 42 (24.0) | 27 (22.1) | 15 (28.3) |  |
| RCA | 78 (44.6) | 60 (49.2) | 18 (34.0) |  |
| PCI in interrogated vessels (%) | 14 (8.0) | 7 (5.7) | 7 (13.2) | 0.128 |
| **Baseline angiographic and physiological parameters** | | | | |
| Minimal diameter, mm | 2.1 (1.8, 2.4) | 2.2 (1.9, 2.5) | 2.1 (1.8, 2.4) | 0.109 |
| Reference diameter, mm | 3.4 (3.0, 3.6) | 3.4 (3.0, 3.6) | 3.3 (2.9, 3.6) | 0.430 |
| Diameter stenosis, % | 35 (29, 40) | 34 (29, 40) | 36 (32, 40) | 0.136 |
| Lesion length, mm | 16.3 (12.2, 23.2) | 16.9 (12.2, 23.2) | 15.2 (12.5, 22.5) | 0.765 |
| Vessel QFR | 0.94 (0.90, 0.96) | 0.94 (0.91, 0.96) | 0.92 (0.89, 0.94) | 0.006 |
| Lesion-specific △QFR | 0.04 (0.02, 0.06) | 0.04 (0.02, 0.05) | 0.05 (0.03, 0.07) | 0.003 |
| **Follow-up angiographic and physiological parameters** | | | | |
| Minimal diameter, mm | 2.1 (1.8, 2.4) | 2.2 (1.9, 2.5) | 2.0 (1.6, 2.3) | 0.001 |
| Reference diameter, mm | 3.2 (3.0, 3.6) | 3.3 (3.0, 3.5) | 3.2 (2.9, 3.6) | 0.357 |
| Diameter stenosis, % | 35 (29, 42) | 33 (28, 41) | 40 (32, 44) | 0.001 |
| Lesion length, mm | 18.4 (13.1, 23.5) | 17.6 (12.6, 23.4) | 19.9 (14.9, 23.5) | 0.207 |
| Vessel QFR | 0.93 (0.88, 0.95) | 0.94 (0.90, 0.96) | 0.90 (0.86, 0.93) | < 0.001 |
| Lesion-specific △QFR | 0.05 (0.03, 0.07) | 0.04 (0.02, 0.05) | 0.06 (0.04, 0.07) | < 0.001 |
| Data are expressed as n (%) or median (25th, 75th percentiles). FP, functional progression; LAD, left anterior descending artery; LCX, left circumflex artery; RCA, right coronary artery; PCI, percutaneous coronary intervention; RWS, radial wall strain; QFR, quantitative flow ratio. | | | | |

| **Supplementary Table 2**. **Univariable** **generalized linear mixed-effects logistic regression analyses: the effect of baseline clinical and angiographic factors on the increase of lesion-specific △QFR** | | | |
| --- | --- | --- | --- |
| Variable | Univariable model | | |
|  | OR | 95% CI | *p*-value |
| Interval time, per month | 0.993 | 0.947-1.041 | 0.771 |
| Age | 1.001 | 0.969-1.034 | 0.947 |
| Male | 0.805 | 0.410-1.580 | 0.526 |
| Acute coronary syndrome | 1.742 | 0.917-3.311 | 0.090 |
| Hypertension | 1.485 | 0.744-2.963 | 0.261 |
| Diabetes mellitus | 2.144 | 1.129-4.071 | 0.020 |
| Previous MI | 1.663 | 0.685-4.033 | 0.259 |
| Dual antiplatelet therapy | 1.595 | 0.803-3.168 | 0.181 |
| ACEI/ARB use | 1.650 | 0.845-3.221 | 0.142 |
| HbA1c | 1.120 | 0.916-1.370 | 0.265 |
| eGFR | 0.977 | 0.958-0.996 | 0.021 |
| Total cholesterol | 0.939 | 0.735-1.200 | 0.613 |
| Triglycerides | 1.095 | 0.858-1.396 | 0.464 |
| High-density lipoprotein cholesterol | 0.578 | 0.165-2.028 | 0.390 |
| Low-density lipoprotein cholesterol | 0.940 | 0.686-1.288 | 0.700 |
| PCI in interrogated vessels | 3.720 | 1.160-11.930 | 0.027 |
| Baseline DS% | 1.014 | 0.971-1.059 | 0.526 |
| Baseline Vessel QFR | 0.943 | 0.893-0.996 | 0.036 |
| Baseline lesion-specific △QFR | 1.022 | 0.935-1.116 | 0.633 |
| RWS_max_ | 1.267 | 1.105-1.453 | 0.001 |
| RWS_max_ > 12 | 3.617 | 1.823-7.176 | < 0.001 |
| OR, odds ratio; CI: confidence interval; MI, myocardial infarction; ACEI, angiotensin-converting enzyme inhibitor; ARB, angiotensin II receptor antagonist; eGFR, estimated glomerular filtration rate; HbA1c, glycosylated hemoglobin; PCI, percutaneous coronary intervention; DS, diameter stenosis; RWS, radial wall strain; QFR, quantitative flow ratio. | | | |
